# ‘Brain Rot’ Among University Students in the Digital Age: A Phenomenological Study

**DOI:** 10.1007/s11920-025-01658-w

**Published:** 2026-02-05

**Authors:** Özkan Özbay

**Affiliations:** Distance Education Application and Research Center, Artvin Coruh University, Artvin, Turkey

**Keywords:** Brain rot, Low-quality digital content, Mental exhaustion, University students, Digitalization

## Abstract

**Purpose of Review:**

This study uses a phenomenological approach to explore how university students perceive and experience “brain rot,” a digital-age phenomenon marked by cognitive decline, attention deficits, and emotional desensitization from prolonged exposure to low-quality digital content.

**Recent Findings:**

Semi-structured interviews were conducted with 15 university students selected through maximum variation sampling, and data were analyzed using Moustakas’ phenomenological method. Findings showed that students associated brain rot with reduced productivity, poor concentration, and impaired decision-making. They reported that low-quality digital content harmed academic performance, caused social isolation, and evoked inadequacy, while coping through self-regulation strategies such as exercise, digital detox, and mindfulness.

**Summary:**

Ultimately, this study provides the first qualitative evidence of how university students experience brain rot as a result of exposure to low-quality digital content and offers a unique and in-depth conceptual framework regarding its impact on individual and academic life in the digital era.

## Introduction

The unprecedented integration of digital media into everyday life has fundamentally reshaped how individuals particularly university students interact, learn, and process information [1]. Frequently described as “digital natives,” these young adults navigate academic, social, and personal domains largely through digital environments, which may increase their susceptibility to certain cognitive and psychological challenges associated with intensive digital exposure [2, 3]. While digital platforms offer notable advantages such as rapid access to information and enhanced social connectivity, a growing body of literature has raised concerns regarding their potential long-term effects on attention span, mental resilience, and executive functioning [4, 5].

Within this evolving discourse, the concept of brain rot has gained increasing academic and cultural attention. Recognized by the Oxford English Dictionary as the Word of the Year in 2024, brain rot broadly refers to perceived cognitive and emotional deterioration associated with prolonged exposure to repetitive, low-quality, and algorithm-driven digital content. Although early philosophical critiques such as Thoreau’s reflections on intellectual superficiality did not explicitly employ this term, contemporary scholarship situates brain rot within digital contexts to describe experiences including diminished attention, impaired memory, emotional blunting, and cognitive fatigue [4, 6, 7].

Empirical studies suggest that sustained interaction with high-frequency digital stimuli, particularly through social media platforms, may undermine users’ capacity for sustained attention, deep reflection, and self-regulation. Digital ecosystems that prioritize immediate gratification such as short-form video platforms tend to reinforce compulsive engagement patterns while simultaneously challenging the cognitive endurance required for academic tasks [8, 9]. In this context, brain rot functions not merely as a colloquial expression but as a conceptual lens through which the psychosocial costs of excessive digital immersion can be examined.

Evidence from cognitive neuroscience further supports the notion that digital saturation may influence neurocognitive functioning. Increased multitasking and reduced tolerance for delayed gratification have been associated with disruptions in executive processes and emotional regulation, particularly among adolescents and young adults whose brains are still undergoing critical developmental changes [10, 11]. For university students who rely heavily on sustained attention, cognitive endurance, and reflective reasoning digital overload may therefore pose challenges to learning efficiency and academic engagement.

Cognitive Load Theory provides a useful framework for interpreting these dynamics. The theory posits that working memory has limited capacity and that excessive exposure to fragmented or non-essential information can impair learning and increase mental exhaustion [12]. In digitally saturated environments, cognitive load is further intensified by continuous partial attention, frequent task switching, and the externalization of memory functions such as reliance on digital tools for information retrieval which may inhibit the development of deep and intrinsic learning strategies [13, 14].

Despite increasing scholarly recognition of the cognitive costs associated with digital media use, the existing literature remains predominantly quantitative, focusing largely on correlations between screen time and performance-related outcomes [4, 15, 16]. What remains comparatively underexplored are students’ subjective experiences specifically how they conceptualize brain rot, recognize its symptoms, and attempt to regain agency over their digital engagement. Recent reviews emphasize that while large-scale surveys and experimental studies provide valuable insights into usage patterns and cognitive outcomes, they often fail to capture the lived, contextualized experiences of young users [17, 18]. The qualitative dimensions of this phenomenon, including emotional responses, coping strategies, and situational influences, are therefore essential for developing theoretically grounded and practically relevant intervention models [19–21].

Accordingly, this study adopts a phenomenological design to explore how university students perceive, experience, and cope with the phenomenon of brain rot. Grounded in semi-structured interviews, the study aims to elucidate how students interpret the cognitive and emotional consequences of prolonged exposure to low-quality digital content. To provide a clear conceptual foundation for this inquiry, Table 1 presents a concise framework outlining the defining characteristics, cognitive, emotional, and behavioral dimensions of brain rot, its theoretical grounding in Cognitive Load Theory, and commonly reported coping strategies. Within this framework, the following research questions guide the inquiry:

**Table 1:** Conceptual framework of the “Brain Rot” phenomenon

Dimension	Description	Key References
Definition	Perceived cognitive and emotional deterioration resulting from prolonged exposure to low-quality, algorithm-driven digital content	Oxford English Dictionary (2024); Wilmer et al. (2017)
Cognitive Effects	Reduced attention span, mental fatigue, superficial information processing, and memory strain	Ward et al. (2017); Calderwood et al. (2014)
Emotional Effects	Emotional blunting, decreased motivation, irritability, and cognitive exhaustion	Montag et al. (2019); Turel & Bechara (2016)
Behavioral Patterns	Compulsive content consumption, frequent task switching, procrastination behaviors	Mark et al. (2008); Kirschner & De Bruyckere (2017)
Theoretical Foundation	Cognitive Load Theory: overload of limited working memory capacity due to excessive and fragmented digital stimuli	Sweller et al. (2011)
At-Risk Population	University students and young adults during sensitive developmental periods	Wilmer et al. (2017)
Coping Strategies	Digital detox, mindfulness-based practices, content limitation, and self-regulation strategies	(Akdeniz Kudubeş et al., 2025; Syvertsen & Enli, 2020).

1. How do university students define and conceptualize the notion of brain rot?
2. What cognitive and emotional effects do students associate with prolonged digital content exposure?
3. What strategies do students report using to cope with or mitigate experiences related to brain rot?

### Literature Review: “Brain Rot” and Digital Media’s Impact on Youth Cognition and Well-Being

“Brain rot” recently spotlighted as the Oxford 2024 Word of the Year refers to the cognitive decline and mental exhaustion purportedly caused by overconsumption of trivial or unchallenging online content [4]. This phenomenon is particularly associated with adolescents and young adults, who often spend excessive time online (e.g. averaging 6–7 h/day on entertainment screen media) consuming low-quality videos, social media feeds, and other digital content [22–25].

Such pervasive digital engagement has raised concerns about deterioration in attention, learning, and mental health colloquially termed “brain rot” in these young populations. Recent research and reviews emphasize that heavy exposure to fragmented, fast-paced online media can lead to shortened attention spans, cognitive overload, emotional desensitization, and habit-forming overuse of screens [4, 26, 27]. For example, instant-gratification platforms like TikTok encourage an endless loop of brief, stimulating content that promotes desensitization and erodes the capacity for sustained attention, making it harder for youth to engage with longer, effortful tasks [7]. Excessive screen immersion also correlates with diminished real-life engagement young people report struggling to enjoy slower-paced activities or in-person experiences after habitual exposure to hyper-stimulating digital media [9, 28]. In summary, “brain rot” encapsulates a pattern of cognitive and emotional effects observed in adolescents and university students immersed in low-quality digital content: poorer attention and memory, reduced motivation for academic or offline pursuits, blunted emotional responses, and increased psychosocial distress [4]. Below, we review recent findings that shed light on these cognitive, emotional, and behavioral impacts, as well as the underlying mechanisms and developmental considerations.

### Cognitive Effects: Attention Deficits and Cognitive Overload

A consistent finding in recent literature is that high levels of digital media use are associated with attention impairments and cognitive overload in adolescents and young adults. The constant influx of online information notifications, messages, scrolling feeds, and multitasking across apps taxes youths’ attentional capacity and working memory. For instance, Li et al. (2022) found that continuous exposure to streaming information (e.g. during video gaming) forces individuals to split attention across multiple stimuli, degrading attentional performance and working memory and even disrupting sleep quality [29]. As the brain is overstimulated by rapid task-switching, youth commonly report difficulty focusing on any single task and describe a “mental cloudiness” or fatigue after long screen sessions [30, 31]. Researchers characterize this as cognitive overload: the brain’s limited capacity for information processing is overwhelmed by the sheer volume and fragmented nature of digital inputs [32, 33]. Mental exhaustion and lapses in memory (so-called “cognitive failures”) have been documented as outcomes of prolonged multitasking and information overload in these contexts [34–36]. Notably, media multitasking such as simultaneously using social media, texting, and watching videos is linked to poorer sustained attention and inhibitory control in college-age students, suggesting that chronic divided attention may undermine the development of concentration skills. Daily diary studies further support this: on days when adolescents engage more frequently with digital media (especially messaging and social apps), they report elevated symptoms of inattention and mind-wandering, even after controlling for baseline attention levels [16, 37]. While moderate use of technology does not appear to severely impair basic cognitive abilities in the general youth population [15, 16], heavy or high-intensity usage patterns (e.g. constant task-switching, hours of scrolling) are associated with small but significant declines in cognitive performance [4]. In particular, executive functions such as working memory, concentration, and decision-making can be weakened under conditions of chronic digital overload. These cognitive effects raise concerns because adolescence is a key period for honing attention control and other executive skills; overwhelming digital input may interfere with this developmental maturation.

### Emotional and Behavioral Consequences

 Beyond cognitive decline, excessive exposure to low-quality digital content also undermines the emotional and behavioral well-being of young people [38, 39]. One prominent effect is emotional desensitization a dulling of emotional responsiveness caused by constant exposure to rapid, high-intensity online media. Adolescents who regularly consume algorithm-driven, sensational content often describe feeling detached or emotionally numb, needing increasingly novel or extreme stimuli to feel engaged [25, 32]. This emotional blunting, often linked to shortened attention spans, weakens empathy and reduces satisfaction with ordinary or offline activities such as reading or face-to-face interaction.

In contrast, certain digital habits intensify negative emotions. Doomscrolling the compulsive consumption of distressing news has been linked to anxiety, stress, and secondary trauma due to continuous exposure to negative content [33, 40]. Likewise, heavy social media use, especially for non-academic purposes, correlates with higher depression and anxiety among college students [23–25]. Yet, it is not merely time online but the quality of engagement that matters most.

Social comparison on platforms like Instagram and TikTok further contributes to emotional strain. Constant exposure to idealized lifestyles fosters negative self-evaluation, body dissatisfaction, and social anxiety [4, 41]. The American Psychological Association (2023) cautions that such comparison lowers self-worth and increases depression risk [42–44]. Over time, reliance on online validation can lead to fragmented identity, insecurity, and social withdrawal [45–47]. Ultimately, overuse of low-quality digital media fosters a cycle of apathy, anxiety, and isolation core features of “brain rot.” Breaking this loop requires fostering emotional resilience and promoting healthier, more mindful digital habits among youth.

### Problematic Screen Use, Reward Cycles, and Neurobiological Mechanisms

Excessive or problematic screen use is increasingly recognized as a behavioral addiction characterized by compulsive engagement with digital media and diminished control over consumption [39, 41]. This pattern is fueled by algorithm-driven reward cycles: social media platforms deliver instant gratification through likes, notifications, and endless content, which sensitizes the brain’s reward system to short-term stimuli [17]. Over time, users become conditioned to seek these immediate rewards, leading to tolerance of constant stimulation and a blunted response to slower, real-life experiences. Research indicates that chronic exposure to fast-paced, “dopamine-driven” digital rewards can foster dependence akin to substance addictions. Neuroimaging studies of heavy internet and smartphone users reveal altered functional connectivity in brain regions involved in cognitive control and reward processing [36], supporting the notion that persistent screen-based stimulation can rewire neural pathways. For instance, adolescents with internet addiction show disrupted communication between prefrontal regulatory circuits and deeper reward centers, correlating with impulsivity and impaired executive function.

Consistent with these findings, a recent review reported that excessive consumption of low-quality online content leads to cognitive overload and emotional desensitization [4]. Affected individuals often experience shortened attention spans, “mental fog,” and difficulty engaging in complex tasks due to continuous partial attention and information overload. At the same time, the cycle of brief rewards and constant novelty can induce emotional numbness and anxiety: users become indifferent to everyday emotional stimuli and may feel restless or anxious when not stimulated by digital media [48, 49]. In summary, problematic screen use perpetuates a self-reinforcing loop of instant rewards and neurobiological changes that undermine sustained attention, self-regulation, and emotional well-being.

### Developmental and Educational Context

The adverse effects of problematic screen use are especially pronounced in adolescents and young adults, for whom critical brain development and educational attainment coincide. Emerging evidence links high levels of screen time in youth to mental health difficulties and developmental risks. For example, a recent study found that university students who spend more than two hours per day on screens (often multitasking across apps) exhibit significantly higher rates of anxiety and depression symptoms compared to peers with lower use [5]. During adolescence and early adulthood, the brain’s executive functions and self-regulatory capacities are still maturing; excessive digital stimulation during this window may disrupt the normal development of attention control, emotional regulation, and working memory [46, 50]. Indeed, prolonged screen exposure has been associated with subsequent deficits in memory and even accelerated cognitive decline in later life, raising concerns about “digital dementia” resulting from formative years spent online [47].

In educational contexts, problematic screen use can erode academic performance and impede learning processes [9]. Students who habitually consume digital content in a fragmented, high-distraction manner often struggle to concentrate on lengthy, complex material. Research has documented that heavy smartphone use correlates with poorer academic outcomes, likely mediated by reduced attention span and increased procrastination [51, 52]. From a cognitive perspective, constant multitasking and rapid task-switching impose a high cognitive load, which interferes with deep processing of educational content [13]. Instead of engaging in critical thinking and reflection, students accustomed to instant online feedback may resort to superficial skimming of information. Overreliance on digital tools (such as search engines and online notes) further encourages externalization of memory individuals remember how to retrieve information online rather than learning the information itself. This phenomenon has been linked to weaker problem-solving skills and lower creativity in young learners [46, 47]. Additionally, by displacing face-to-face interactions, uncontrolled screen use can impair socio-emotional development and student well-being. In sum, the developmental stage and academic environment make youth particularly vulnerable to the adverse effects of pervasive screen time. Educators and clinicians are increasingly concerned that without intervention, today’s digital-native students may experience long-term impairments in cognitive development, mental health, and educational success.

## Methods

### Design

This study adopted a phenomenological design to explore how university students define and experience brain rot. Phenomenology focuses on individuals’ lived experiences and subjective meanings [53, 54].

### Participants

The study included 15 undergraduate students from the Faculties of Education, Health Sciences, Arts and Sciences, and Engineering at a public university. Using maximum variation sampling [55], participants with diverse academic backgrounds and high digital content use were selected. Eligibility criteria included owning a smartphone for at least one year, regular mobile internet use, and at least one hour of daily digital content consumption. Semi-structured interviews were conducted until data saturation was reached. Participants’ mean age was 23 (SD = 1.38), and their socio-demographic and digital use characteristics are detailed in Table 2.

**Table 2:** Socio-demographic characteristics, digital usage habits, and health status of the participants

Variables			*n*	%
Demographic Information	Age	21–23 24–26	9 6	60.0 40.0
	Gender	Female Male	7 8	46.7 53.3
	Faculty of study	Education Health Sciences Arts and Sciences Engineering	4 4 4 3	26.6 26.6 26.6 20.2
	Year of study	1st year 2nd year 3rd year 4th year	3 4 4 4	20.2 26.6 26.6 26.6
Digital Usage Habits	Daily digital use (hrs)	1–2 h 3–5 h 6–8 h 8 h or more	1 7 6 1	6.7 46.7 40.0 6.7
	Most frequently used digital platforms*	WhatsApp Instagram YouTube TikTok	15 14 9 3	100.0 93.3 60.0 20.2
	Primary device used to access digital platforms*	Phone Computer Tablet	15 8 5	100.0 53.3 33.3
Health Information	Average daily sleep duration	4–6 h 7–8 h More than 8 h	8 6 1	53.3 40.0 6.7
	Engagement in regular physical activity	Yes No	8 7	53.3 46.7
	Presence of a diagnosed mental health condition	Yes No	0 15	0.00 100.0

*Multiple responses were selected.

### The Research Team and Reflexivity

The researcher, holding a PhD in Computer and Instructional Technologies Education, has extensive experience in qualitative research and digital learning. Their background enabled a deep understanding of students’ digital behaviors and fostered empathy and trust during interviews. Reflexivity was maintained throughout the study, with ongoing reflection on personal biases and their potential influence on data interpretation.

### Procedures

Semi-structured, face-to-face interviews were conducted with 15 students in a private campus office. The interview guide, developed through a literature review and pilot testing, included open-ended questions (Appendix 1). Each interview lasted 30–45 min. Participants provided informed consent, and all sessions were audio-recorded with permission. Confidentiality and ethical principles of voluntariness and anonymity were strictly upheld.

### Data Analysis

Interviews were transcribed verbatim and analyzed using Moustakas’ (1994) phenomenological method. Meaningful statements were coded, clustered into subthemes and themes, and analyzed using MAXQDA 2020 to support conceptual mapping and visualization. Textural and structural descriptions were developed to capture both experiences and their contextual meanings. Credibility was enhanced through member checking, expert review, and transparent reporting. Ultimately, five main themes and several subthemes were identified, illustrating the essence of the brain rot phenomenon.

## Result

The average age of the students who participated in the study was 23.2 ± 1.38 years. Of the participants, 53.3% were male. In terms of academic background, 26.6% were enrolled in the Faculty of Education, 26.6% in the Faculty of Health Sciences, 26.6% in the Faculty of Arts and Sciences, and 20.2% in the Faculty of Engineering.

Regarding daily digital device usage, 46.7% of participants reported spending 3–5 h per day engaged with digital content. Additionally, 53.3% stated that they sleep between 4 and 6 h daily, and the same percentage reported engaging in regular physical activity. None of the participants had any diagnosed mental health conditions.

**Fig. 1:**
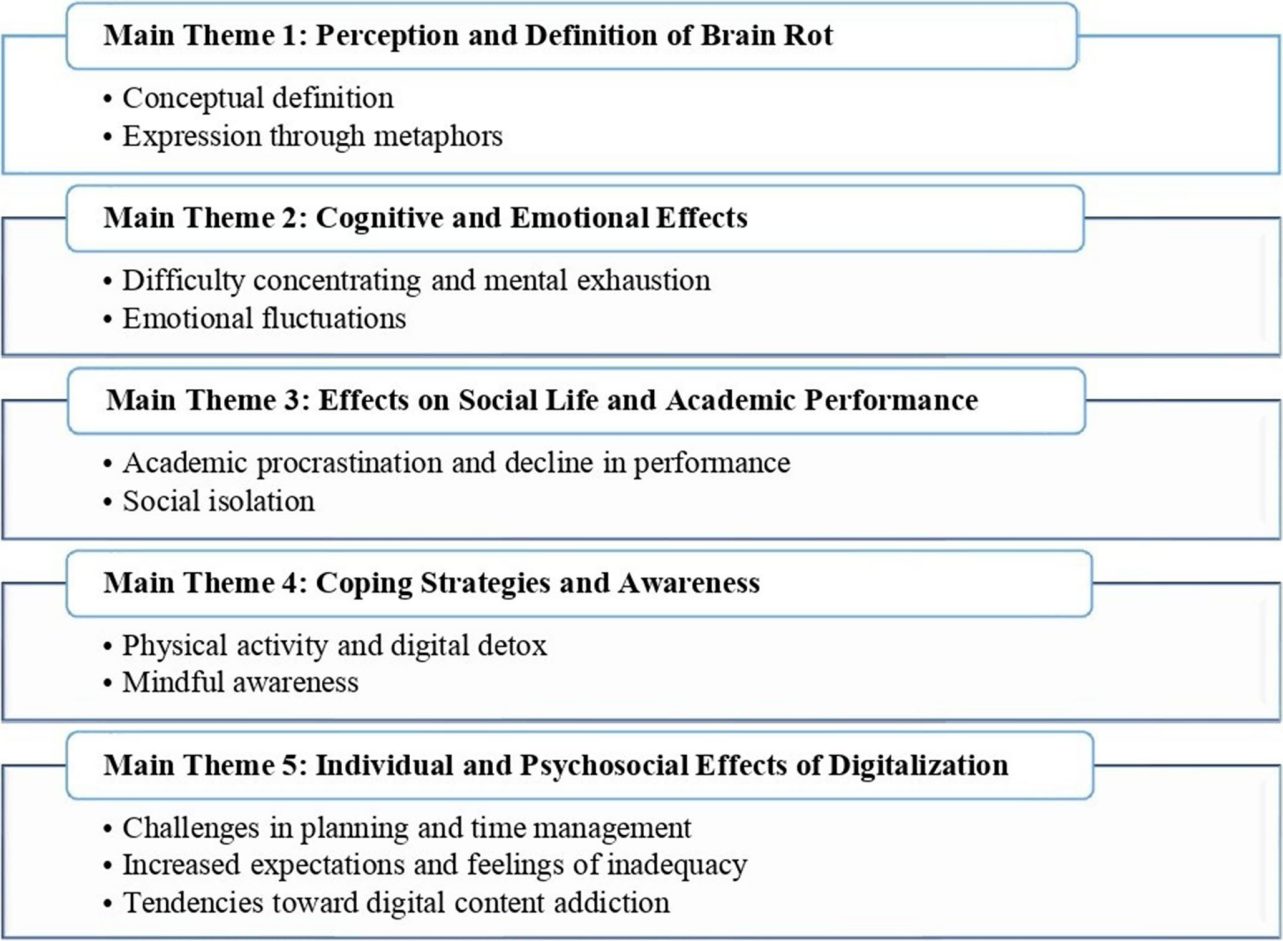
Themes and sub-themes

Based on participants’ views on the concept of *brain rot* and digital content consumption, five main themes and eleven subthemes were identified (Fig. 1).

### Main Theme 1: Perception and Definition of Brain Rot

This theme encompasses students’ definitions, personal interpretations, and experiences related to the concept of brain rot. Participants commonly described the condition in terms of decreased productivity, mental fog, and difficulty in making decisions.

### Subtheme 1.1: Conceptual Definition

Participants interpreted *brain rot* as a concept through their own experiences and noted that this condition has become inevitable in the digital age.

*“For me*,* brain rot means a decline in perception resulting from prolonged exposure to digital content*,* which negatively affects one’s ability to comprehend and process information.” (P10)*.

*“My mind can no longer focus on anything because of the constant content exposure. My thoughts are blurry*,* and I have difficulty making decisions. It’s like my brain is full but can’t process anything.” (P3)*.

*“Brain rot isn’t just about losing the ability to think—it also feels like emotional numbness. I’m experiencing a kind of inner stagnation*,* even burnout.” (P7)*.

### Subtheme 1.2: Expression Through Metaphors

The concept of brain rot was expressed by participants through various metaphors. These metaphors vividly illustrate the deterioration of cognitive functions using symbolic imagery.

*“Like a static-filled television. It seems to be working from the outside*,* but the image is blurry. My brain becomes like that after a certain point.” (P10)*.

*“For me*,* it’s like a drained battery. After spending a long time on social media*,* my energy is gone and I feel empty.” (P13)*.

*“Like a rusting piece of iron. My thoughts move slowly*,* and I feel immobilized.” (P7)*.

*“Like ideas falling into a dark*,* infinite void. Brain rot swallows my thoughts.” (P2)*.

*“Like an overstuffed wardrobe full of clothes that don’t match the season. My brain is filled with useless information and becomes dysfunctional.” (P14)*.

*“Like an old car in need of maintenance. My mind slows down and my reactions are delayed.” (P15)*.

### Main Theme 2: Cognitive and Emotional Effects

This theme addresses the impact of low-quality content consumption on university students’ cognitive processes and emotional balance. Among the most prominent effects are concentration difficulties, emotional fluctuations, and increased stress.

### Subtheme 2.1: Difficulty Concentrating and Mental Exhaustion

Participants reported that increased screen time leads to distraction and mental exhaustion.

*“When I sit down to study*,* I get distracted immediately. It feels like my mind is already full of other things.” (P9)*.

*“I struggle to watch long-form content like movies or series now. I just can’t keep my attention focused.” (P5)*.

*“My energy drops*,* and I feel like I’m in a void. No matter what I do*,* I can’t concentrate.” (P3)*.

### Subtheme 2.2: Emotional Fluctuations

Feelings of stress, anxiety, and dissatisfaction were commonly reported following intense consumption of low-quality digital content.

*“My stress levels increase. I feel tired and inadequate.” (P11)*.

*“At first*,* digital content makes me feel good*,* but then I’m left with a sense of emptiness.” (P1)*.

*“Sometimes I feel energetic*,* but most of the time*,* the feeling of unhappiness dominates.” (P13)*.

### Main Theme 3: Impact on Social Life and Academic Performance

This theme explores the negative effects on students’ academic performance and social relationships. Low-quality digital content leads students to withdraw from face-to-face social interactions and contributes to procrastination in their academic responsibilities.

### Subtheme 3.1: Academic Procrastination and Decline in Performance

The majority of participants stated that low-quality digital content hinders their ability to study and negatively affects their academic performance.

*“I constantly postpone studying. It’s hard to start because digital content is more appealing.” (P10)*.

*“Even though my academic performance hasn’t dropped drastically*,* my inability to focus slows down my learning process.” (P5)*.

*“I can’t make time for classes because I’m always watching something on my phone.” (P13)*.

### Subtheme 3.2: Social Isolation

The dominance of low-quality digital content over social interactions leads to a weakening of friendships and social connections.

*“We gather together*,* but everyone is on their phone. We don’t even talk.” (P14)*.

*“Because of social media*,* my face-to-face interactions have decreased.” (P1)*.

*“Since I spend so much time online*,* I devote less time to my social circle.” (P9)*.

### Main Theme 4: Coping Strategies and Awareness

Participants stated that they had developed various coping strategies to overcome the negative effects of *brain rot*. Physical activity, digital detox, and cultivating mindfulness were frequently emphasized approaches.

### Subtheme 4.1: Physical Activity and Digital Detox

Participants reported engaging in physical activities such as walking and spending time in nature to distance themselves from the negative effects of low-quality digital content and achieve mental relief. They also practiced digital detox strategies by limiting screen time for specific periods.

*“I go for walks or spend time with my friends. I try not to look at my phone.” (P12)*.

*“When I engage in physical activity*,* my energy increases and my mind relaxes.” (P7)*.

*“When I stay off social media for a while*,* I feel more productive and refreshed.” (P10)*.

### Subtheme 4.2: Mindful Awareness

Participants turn to mentally calming activities such as reading books and spending time in nature.

*“I relax my mind by reading books.” (P14)*.

*“I’ve made it a habit to spend a few quiet minutes in the morning instead of checking my phone right away. It makes me feel like I’m in control of my day.” (P5)*.

*“Now I question how time passes when I’m in front of a screen. Just becoming aware of what I’m watching or why I’m watching it has helped me act more mindfully.” (P12)*.

### Main Theme 5: Individual and Psychosocial Effects of Digitalization

This theme encompasses the multifaceted effects of digitalization on university students’ individual and psychosocial well-being. Participants reported that digitalization has led to various challenges, pressures, and transformations, particularly in their academic and social lives.

### Subtheme 5.1: Challenges in Planning and time Management

Low-quality digital content hinders students’ ability to study in a planned manner and makes time management difficult.

“I keep postponing my planned tasks because of digital distractions. My time management has deteriorated.” (P6).

“I’ve lost my sense of time and can’t stick to my schedules.” (P12).

“Due to digital content, I’m staying up late and waking up tired in the morning.” (P13).

### Subtheme 5.2: Increased Expectations and Feelings of Inadequacy

Participants expressed that the content they encounter on social media creates feelings of inadequacy within themselves.

*“Seeing content where everyone appears happy and productive makes me feel like I’m falling behind.” (P13)*.

*“My expectations have increased*,* but I can’t meet them*,* which causes me more stress.” (P11)*.

*“The digital world sometimes distorts reality. Distancing from real life creates a feeling of inadequacy.” (P14)*.

### Subtheme 5.3: Tendencies Toward Digital Content Addiction

Some participants expressed that low-quality digital content consumption is not merely a habit but has evolved into an addictive behavior. Particularly notable are feelings of restlessness and deprivation when away from content, along with repeated urges to return to it.

*“Staying away from digital content is as hard for me as quitting smoking. When I’m away*,* I feel withdrawal. These contents are easily accessible but cause serious harm in the long run.” (P15)*.

*“I feel restless when I don’t have my phone in hand. It feels like I’m constantly missing out on something.” (P9)*.

*“I don’t know what to do without digital content. When I’m away from it*,* I feel empty.” (P3)*.

## Discussion

This study deeply explored university students’ perceptions, experiences, and coping strategies related to the concept of brain rot using a phenomenological approach. The themes identified align with conceptual discussions in the existing literature and provide qualitative evidence of the multifaceted effects of digital content on individuals’ cognitive and emotional functioning. The absence of qualitative studies addressing the brain rot concept based on university students’ subjective experiences highlights the originality of this research and its significant contribution to the literature.

### Theme 1: Perception and Definition of Brain Rot

In recent years, increasing academic interest in the effects of digitalization on individuals’ cognitive functions has made the concept of brain rot a noteworthy but still relatively underexplored area of discussion. This concept is primarily used to describe the impairment in executive functions caused by continuous and intense exposure to low-quality digital content [4].

Participants in this study commonly associated the concept of brain rot with mental fog, distraction, and difficulty in decision-making. These experiences align with the literature describing reduced attention span, information overload, and resulting burnout symptoms (Firth et al., 2019). Furthermore, it has been reported that constant exposure to algorithm-driven content flows on platforms such as social media reduces individuals’ ability to engage with meaningful information and increases anxiety levels [56].

Brain rot is directly linked to digital behavioral patterns like doomscrolling, which are prevalent especially among young individuals. Such behaviors are reported to lead to distraction, mental fatigue, and cognitive exhaustion [8, 48]. The participants’ views support these findings, indicating that brain rot is not merely a subjective perception but is experienced as one of the characteristic cognitive and emotional symptoms of the digital age.

### Theme 2: Cognitive and Emotional Effects

The second theme, cognitive and emotional effects, underscores how prolonged exposure to low-quality digital content impacts university students’ mental functions and emotional balance. As digital natives, Generation Z and Alpha have grown up in constant contact with screens, making digital engagement an integral part of daily life [10]. However, this continuous exposure strains executive functions, attention control, and decision-making, often leading to mental exhaustion or “brain rot” [4, 57].

Research shows that rapid and fragmented digital content overloads working memory and depletes cognitive resources, while media multitasking diminishes focus and performance in deep-thinking tasks [4, 58]. Participants in this study described mental fog, indecisiveness, and reduced productivity when frequently consuming low-quality content [32, 33].

Emotionally, constant digital stimulation fosters desensitization, loss of empathy, and emotional exhaustion [4]. Social media algorithms promoting instant gratification condition users’ reward systems to seek quick pleasure, causing apathy and emotional numbness in real-life interactions [17]. Feelings of inner emptiness, boredom, and anxiety emerge within this cycle. Additionally, doomscrolling heightens stress, anxiety, and secondary traumatic symptoms, while social media absence triggers FoMO [33, 48].

Overall, brain rot among university students extends beyond attention deficits to include emotional fatigue, anxiety, and motivational loss. These findings reveal the dual cognitive emotional burden of digital media, posing significant risks for academic performance and long-term psychological well-being.

### Theme 3: Effects on Social Life and Academic Performance

Participants in this study reported that exposure to low-quality digital content negatively affects not only their cognitive and emotional processes but also their academic performance and social relationships. Consistent with digital addiction research, excessive digital use contributes to attention fragmentation, social isolation, and academic procrastination [10].

Academically, heavy consumption of low-quality content weakens students’ focus and comprehension, especially in tasks requiring sustained attention and critical thinking [34]. Frequent multitasking, managing notifications, and switching between platforms disrupt the continuity of learning and lead to cognitive fatigue, reducing academic motivation and productivity [4, 51]. The resulting impairments in executive functions and working memory make it difficult for students to perform well on exams and assignments, reflecting the cognitive dimension of brain rot.

Socially, prolonged engagement with digital media limits genuine interaction and fosters behaviors like phubbing, where individuals ignore those around them to engage with their devices [59]. Participants described decreased empathy, poor listening, and weakened conversational skills, leading to loneliness and disconnection [18, 41]. Social media’s constant stream of idealized content fuels comparison and inadequacy, while temporary digital rewards such as likes and comments promote superficial interactions over meaningful relationships [17, 20, 60].

In sum, low-quality digital content consumption undermines both academic performance and social connectedness. The combined effects of distraction, procrastination, and diminished real-life engagement illustrate how brain rot exerts a pervasive influence on young adults’ learning and interpersonal well-being.

### Theme 4: Coping Strategies and Awareness

Findings indicate that university students adopt various self-regulation strategies to mitigate the effects of brain rot and maintain mental well-being. Commonly reported methods include digital detox, mindfulness, and physical activity, reflecting a growing sense of digital awareness [19, 21, 61].

Digital detox involves intentionally limiting time spent on digital platforms to restore focus and reduce mental fatigue. Studies show that even short-term detox interventions can alleviate anxiety and depressive symptoms in young adults [21, 62]. Similarly, mindfulness-based practices enhance attention control and help individuals engage with technology more consciously, promoting sustained cognitive balance [29, 63].

Participants also reported practical attention-control strategies such as disabling notifications, deleting distracting apps, and using digital media with purposeful intent behaviors consistent with “mindful technology use.” Additionally, engaging in offline activities such as walking, reading, and socializing provides emotional fulfillment and reduces digital fatigue [63].

Overall, maintaining mental health in the digital era depends on awareness-driven self-regulation and integrating these strategies into daily routines. Educational institutions can play a key role by organizing digital literacy workshops, mindfulness sessions, and time management training to support students in building healthier digital habits.

### Theme 5: Individual and Psychosocial Effects of Digitalization

Findings reveal that digitalization has profoundly transformed university students’ social lives, learning habits, and psychological well-being. While offering easy access to information and flexible learning environments, it also brings challenges such as poor time management, social comparison, and digital dependency [8, 13]. Constant multitasking and exposure to digital stimuli deplete cognitive resources, promote superficial learning, and hinder deep information processing [10]. Participants frequently reported difficulty focusing and managing study time effectively.

Socially, digitalization presents both opportunities and drawbacks. Although online platforms facilitate communication, they simultaneously weaken face-to-face connections and increase social isolation [22]. Continuous online engagement fosters behaviors like phubbing and diminishes empathy and belonging [59].

Moreover, dopamine-driven rewards such as likes and notifications reinforce dependence on digital validation, contributing to impatience, shallow attention, and dissatisfaction with offline life [35, 36]. Reliance on digital memory systems also weakens critical thinking and creativity by promoting recall of information sources rather than analytical engagement [46, 47].

In conclusion, digitalization exerts both educational benefits and psychosocial risks. Universities should adopt balanced strategies that foster deep learning, self-regulation, and authentic social connection while encouraging mindful and responsible digital engagement.

### Limitations

This study has several limitations that should be considered when interpreting the findings. First, the participants were recruited from a single public university, which may limit the transferability of the results to students from different geographical regions, cultural contexts, or educational institutions. Although maximum variation sampling was used to capture diverse academic backgrounds, the findings should be interpreted as context-specific rather than representative of all university students.

Second, data were collected exclusively through semi-structured individual interviews. While this approach allowed for in-depth exploration of participants’ lived experiences, it may have constrained the emergence of shared meanings, contrasting viewpoints, or interaction-driven insights that could be elicited through focus groups or other collective qualitative methods. Future studies employing mixed qualitative designs may provide a more comprehensive understanding of the phenomenon.

Third, the study relied on participants’ self-reported perceptions and experiences of low-quality digital content consumption. As a result, objective indicators of digital media use such as actual screen time, application usage logs, or behavioral tracking data were not incorporated. This reliance on subjective reporting may introduce recall bias or socially desirable responses and limits the ability to directly link perceived experiences with measurable digital usage patterns.

Finally, given the qualitative and phenomenological nature of the study, the findings are not intended to establish causal relationships between digital content exposure and cognitive or emotional outcomes. Rather, they offer an in-depth exploration of how university students subjectively experience and interpret the phenomenon of brain rot. Future research is encouraged to build on these findings by employing longitudinal, experimental, or mixed-method approaches, incorporating larger and more diverse samples, and integrating objective digital behavior measures to strengthen explanatory and causal inferences.

## Conclusion

This phenomenological study explored how university students perceive and experience the phenomenon of brain rot in the digital age. Participants commonly described brain rot as a subjective state characterized by cognitive and emotional strain, including difficulties in concentration, reduced perceived productivity, and feelings of emotional desensitization. Students associated these experiences with prolonged engagement with low-quality digital content, particularly in relation to their academic focus and social engagement.

Participants also reported employing various self-regulation strategies such as digital detox practices, mindfulness, and physical activity to cope with these experiences and to regain a sense of cognitive balance and emotional control. While these strategies reflect students’ adaptive efforts rather than established interventions, the findings suggest that increasing awareness of mindful technology use and self-regulatory skills may be beneficial. In this context, higher education institutions may consider developing supportive initiatives aimed at fostering digital awareness and promoting reflective and balanced engagement with digital technologies, while acknowledging that further research is needed to evaluate the effectiveness of such approaches.

## Data Availability

No datasets were generated or analysed during the current study.

## Appendix 1: Interview Questions

1. What does the concept of “Brain Rot” mean to you based on your own experiences or observations? Could you please share your definition of this concept?
2. If you had to explain the concept of “Brain Rot” using a metaphor, what would that metaphor be? Please explain in detail why you chose this metaphor.
3. How do you think the intensive consumption of low-quality digital content is connected to the concept of “Brain Rot”?
4. What effect do you think intensive consumption of low-quality digital content has on you?
5. When you consume low-quality digital content intensively, which types of content make you feel mentally tired or exhausted?
6. How are your daily activities affected when you consume low-quality digital content intensively?
7. How does intensive consumption of low-quality digital content affect your academic performance?
8. How would you evaluate the impact of intensive consumption of low quality digital content on your social relationships?
9. How would you evaluate the impact of intensive consumption of low quality digital content on your self-care routines?
10. How do you cope with the “Brain Rot” that arises from intensive consumption of low-quality digital content?
11. If you imagined spending a day without digital devices, how would this affect your mental state? What kind of experience would it be for you, and how would you feel mentally?
12. How do you think intensive consumption of low-quality digital content poses a problem for university students?
13. Is there any other thought or experience related to this topic that you would like to add?
